# China's Response to the 2014 Ebola Outbreak in West Africa

**DOI:** 10.1002/gch2.201600001

**Published:** 2017-01-30

**Authors:** Yanzhong Huang

**Affiliations:** ^1^ Center for Global Health Studies, School of Diplomacy and International Relations Seton Hall University South Orange NJ 07079 USA

**Keywords:** China, Ebola, global health governance, global health security, public health emergencies of international concern

## Abstract

Beginning in March 2014, West Africa has endured the largest outbreak of Ebola viral disease (EVD) in history. The crisis highlighted the role of China in addressing public health emergencies of international concern (PHEIC). Through bilateral and multilateral channels, China kicked off its largest ever humanitarian mission in addressing a PHEIC. The unprecedented generosity served the domestic needs to prevent EVD from spreading into China, but it was also consistent with China's foreign policy objective to pursue soft power in Africa. While its total funding to EVD control in West Africa was no match of top donors like the United States, it becomes much more impressive when adjusted for gross domestic product (GDP) per capita. As Beijing becomes more sensitive to disease outbreaks overseas and as the scope of its humanitarian engagement grows and diversifies, the space for China's cooperation with international actors over global health governance is expected to further expand.

## 1 Introduction

Beginning in March 2014, West Africa has endured the largest outbreak of Ebola viral disease (EVD) in history. As of June 2016, the virus has infected over 28 600 people and caused more than 11 300 deaths.^1^ The catastrophe brought to light “massive failure of global health governance.”^2^, ^3^ But the same crisis also seemed to highlight the role of China in addressing public health emergencies of international concern (PHEIC).^4^ While visiting West Africa in August 2015, Chinese foreign minister Wang Yi noted that China in carrying out its largest ever health aid program in history created multiple “firsts”: the Chinese President was the first head of state to commit explicitly to answering the call for help by three Western African countries; China used large chartered airplanes to ship the first batch of badly needed anti‐epidemic supplies; China for the first time deployed a whole unit of epidemic prevention forces and military medical staff abroad; China built a Biosafety Level 3 (BSL‐3) lab overseas, and set up an infectious disease medical center in another country for the first time.^5^

While the accuracy of Wang's remarks is debatable, they raise interesting questions regarding China's involvement in the global response to the EVD outbreak. How responsive has China been in addressing the outbreak? What factors have incentivized China to respond in the way that it did? Did China contribute its fair share? And to what extent does this tell us about China's engagement in future global governance for public health emergencies? Using both quantitative and qualitative data, this paper examines the patterns and performance of China's response to the 2014 Ebola outbreak. The study finds that Beijing has become increasingly sensitive to disease outbreaks that originate from abroad, and the expanding humanitarian engagement opens the space of international cooperation over the global governance for PHEIC.

## 2 China's Domestic Response

Unlike its initial mishandling of the 2002–2003 Severe acute respiratory syndrome (SARS) outbreak, the Chinese government's response to the Ebola outbreak was swift and proactive. On March 24, 2014, right after Guinea health authorities confirmed the presence of an EVD outbreak, the Chinese embassy sent warnings to Chinese nationals there. In early April, the Chinese government provided Guinea with $50 000 in cash as emergency humanitarian aid.^6^ This was followed by emergency assistance to the two other Western African countries most severely impacted by the outbreak, Liberia and Sierra Leone. At that time, China claimed that it was the first country to provide Sierra Leone medical aid to fight Ebola.^7^

Almost at the same time, China began to undertake Ebola prevention and control measures back home. Emphasis was placed on monitoring passengers arriving from Ebola‐affected countries for fevers. In Guangzhou, which has up to 200 000 African residents,^8^ the local government authorities implemented de facto quarantine measures against passengers from West Africa, who were asked to stay at designated hotels and report their body temperature at required intervals using Global positioning system (GPS)‐enabled mobile phones.^9^ On August 12, 4 d after the World Health Organization (WHO) declared the EVD outbreak to be a PHEIC, China set up a joint prevention and control mechanism consisting of 22 central ministries to address Ebola prevention and control and to develop a contingency plan for the outbreak.^10^ By November, the mechanism had enabled the health and inspection departments to screen more than 26 000 arrivals, identifying 88 people with fevers, though no confirmed cases of Ebola were found.^11^

The outbreak also prompted China to accelerate the development of Ebola diagnostic kits and medical countermeasures. In August, the People's Liberation Army (PLA) academy of military medical science announced that it had developed China's first drug (jk‐05) for treating Ebola. In November, the Chinese food and drug administration approved the test reagent developed by three Chinese firms, making China one of the few countries that could produce diagnostic kits. The same month, the national health and family planning commission (NHFPC) distributed clinical guidelines on Ebola response to all local health administrations and hospitals in China.^12^

Yet compared to its high‐profile responses to the 2009 H1N1 pandemic and the 2013 H7N9 outbreak, China seemed to adopt a less aggressive domestic approach toward the 2014 EVD outbreak. In sharp contrast to the zealous coverage of the previous two flu outbreaks, Chinese media and society appeared nonchalant toward the possible spread of Ebola in China.^13^ Between August and December of 2014, the author had three times visited China, but did not notice any apparent panic or fear among ordinary Chinese over the potential spread of Ebola in China.

The relatively low‐profile domestic response was not so much the result of growing confidence in the government response capacity as it was the result of a more realistic assessment of the risk that EVD posed to China. A cross‐sectional survey carried out in Guangzhou in November 2014 found that more than half of the respondents lacked confidence in the government ability to control an EVD outbreak.^14^ Earlier, a study conducted by Chinese scientists predicted a total of 6 to 194 imported cases in China.^15^ In view of the growing trade and travel links, some leading international public health experts were concerned that China was at serious risk of Ebola.^16^ Senior Chinese health officials agreed that there was risk of the virus spreading into China or Chinese citizens contracting the virus overseas, but they apparently had no intention to overestimate the risk and incite major worries at home.^4^, ^17^ After all, China did not have any direct flights to the three Western African countries. According to a study published in the *Lancet*, the top six destination countries of flights departing from West Africa were all in other parts of Africa followed by three European countries. China was ranked number ten in terms of passenger destinations, but the total passenger flow only accounts of 18% of that of UK and France combined.^18^, ^19^

That being said, and the perceived low risk of Ebola spread did not lead China to write off the threat of the virus completely. The EVD outbreak unfolded at a time when the Youth Olympic Games was about to be held in Nanjing and a large number of Africans were anticipated to visit China in late August.^20^ Noting, “a single Ebola case during the games would become headlines overshadowing news on the games,” an editorial of the official *Global Times* on August 4 called for “doing a seamless job in preventing the virus from infiltrating China.”^20^ There were also concerns that the Ebola virus might ruin the annual World Economic Forum meeting in the city of Tianjin, where hundreds of business and research leaders were to gather in September, and the 2014 Asia‐Pacific Economic Cooperation Economic Leaders' Meeting, which was to be held in Beijing in November. The Chinese Center for Disease Control and Prevention (China CDC) vowed to implement “Olympic‐level preventative measures” to contain the virus.^21^ In mid‐August, when the Youth Olympics began, the government kicked off new preventive measures, including banning athletes from affected countries from competing in certain events.^22^

The EVD outbreak in West Africa also threatened Beijing's economic interests in the region. China is Africa's largest trade partner and Chinese firms currently engage in $15 billion worth of work in West Africa. It is a key trading partner for the three most affected countries, accounting for 47% of Sierra Leone's total trade volume, 18% of Liberia's, and 12.4% of Guinea's.^23^ Prior to the outbreak, China's growing economic activities with Africa had raised the question of whether Beijing was only interested in accessing the market in order to secure the supply of natural resources in Africa and not in promoting inclusive economic development in the subcontinent. During the EVD outbreak, there were reports suggesting that China's efforts to develop mineral deposits and build roads in Western Africa might have brought infected animals into closer contact with humans, thereby facilitating the rapid spread of Ebola.^24^ For economic and reputational reasons, it was in Beijing's interest to improve its image in the region. Besides participating actively in peace building efforts, health aid had increasingly become an important foreign policy instrument for China to project in Africa its soft power, defined by Joseph Nye as the ability to shape the preferences of others through appeal and attraction.^25^, ^26^, ^27^ As the Second White Paper on Foreign Aid indicated, China emphasized the delivery of emergency humanitarian aid as a major form of China's foreign aid.^28^ The Ebola outbreak thus opened a window of opportunity for China to showcase its soft power in Africa while foiling criticism about Beijing's mercantilist behavior in the region.^29^ China made no secret its intention to use the humanitarian aid as a tool to compete with other countries for soft power in Africa. According to a scholar at the government think tank:

As a matter of fact, China's soft power building in Africa is still at its preliminary stage. In particular, China suffers from the deficit of cultural soft power in Africa. China's advantages in soft power building in Africa lies more in the attractiveness of its African policy, that is, foreign policy, in which health diplomacy to Africa as an important component of public diplomacy is the most effective and most influential.^30^

Against this background, it came as no surprise that the front line of China's battle against Ebola was moved beyond its borders. According to senior Chinese health officials, providing aid to West Africa was crucial in China's efforts to construct a “barrier” against the spread of the virus.^31^ Minister Li Bin of NHFPC was candid when she explained why China supported Western Africa's fight against Ebola: “Infectious diseases know no boundaries … China by helping West African nations is also helping itself.”^32^

## 3 China's Humanitarian Assistance

Overall, China responded to the Ebola epidemic in West Africa with unprecedented generosity. While it was not unusual for China to offer humanitarian aid to countries affected by natural disasters, this was the first time China extended massive humanitarian aid to countries fighting a public health emergency. By late November of 2014, China had—throughout four consecutive phases in April, August, September, and October—offered $123 million (750 million *yuan*) worth of humanitarian aid to the global Ebola control efforts, China's largest ever response to an international humanitarian crisis. The package included the provision of in‐kind contributions comprising of ambulances, motorcycles, medical equipment as well as prevention care supplies. It also included food aid, deployment of medical teams, and public health experts, as well as labs and treatment centers. Unlike other donors, China also provided aid to countries surrounding the three Western African countries: it provided 1800 tons of equipment and supplies to 13 countries in the region.^33^ In Guinea, two‐thirds of the anti‐Ebola supplies were reportedly from China. There were also reports that by mid‐October China had sent enough of the experimental anti‐Ebola drug (jk‐05) to West Africa to treat 10 000 people.^34^ While the effectiveness of the drug remains unknown, another Chinese‐developed experimental drug (MIL77) successfully treated a British military nurse who contracted Ebola while serving in Sierra Leone.^35^ Chinese scientists also developed their own Ebola vaccine that was found to be effective based on results of the first phase‐one clinical trial.^36^ Unlike the other two experimental vaccines in human trials developed by the U.S. and Canadian scientists, the Chinese vaccine is the only one that has used genetic material from the current outbreak strain.^37^ In May 2015, the WHO also approved an Ebola test reagent developed by a Chinese firm, which was ten times more sensitive than the benchmark for existing Ebola diagnostic.^38^

China dispatched a large number of health personnel to West Africa. In August 2014, it sent three teams of infectious disease experts (totaling 115 people) to assist local medical professionals in the Ebola‐stricken countries. This occurred at a time when aid groups from the United States, Europe, and Japan were evacuating their own in droves.^29^ In mid‐September, a 59 member Chinese laboratory team departed for Sierra Leone to help the country build its lab testing capacity, joining the Chinese medical staff that had been on the ground virtually since the beginning. The WHO Director‐General Margaret Chan called China's commitment “a huge boost, morally and operationally.”^39^

In November, China launched a treatment center in Liberia. According to the Chinese ambassador to Liberia, China was the only country to provide the construction of an Ebola treatment unit (ETU) as well as the operation and staffing of the unit.^40^ This was followed by the announcement that China would send an additional 1000 personnel to help fight the outbreak, making China the largest contributor of medical staff to the crisis.^41^ In Sierra Leone, the mobile lab that China helped construct reportedly tested 20% of the samples in the country, with 100% accuracy. In the meantime, China started to build a permanent biosafety lab in Sierra Leone. Launched in March 2015, the lab was the first permanent BSL‐3 lab in Africa. By the end of the month, Chinese health personnel had tested 5000 samples and treated 900 patients in West Africa.^42^ The number of Chinese professionals would eventually reach 1200.^5^ In addition, Chinese health workers trained 12 000 local medical and public health personnel.^43^

Despite its preference for bilateral aid, some of China's aid to Western Africa was routed through international and regional organizations. In October 2014, China pledged to provide $6 million to the World Food Programme (WFP) for vital food supplies, as well as $2 million funding for WHO and the African Union respectively. Through WFP, China provided 5500 tons of food aid to the three most affected Western African countries.^33^ On December 2, China contributed an additional $6 million to complement UN emergency efforts through the UN Ebola Response Multi‐Partner Trust Fund.^44^

Besides tangible assistance, Chinese domestic experience in fighting major disease outbreaks offered a reference point for the three countries most affected by the Ebola virus. Since the virus is transmitted through contact with bodily fluids, quarantine measures—widely used in China's fight against avian flu—could help break the chain of infection. Given that early symptoms of Ebola infection are similar to those of H7N9, China's experience in handling the flu cases could also be useful to Ebola treatment. In August 2014, the official Xinhua News Agency quoted WHO officials saying that African countries could learn from China's experience in addressing the 2013 H7N9 outbreak as well as from its successful investment in public health.^65^ A Chinese scholar went as far as to claim that, “China's experience [in disease prevention and control] applies to the whole world.”^45^ There were reports suggesting that Liberia did learn from China in applying some of the public health measures such as quarantine to Ebola control.^46^

## 4 Did China Contribute its Fair Share?

The role of major non‐state actors such as Médecins Sans Frontières (MSF) notwithstanding, China appeared to be more responsive to the EVD outbreak in West Africa than many The Organization for Economic Cooperation and Development (OECD) countries in the initial stage of the crisis. In April and August 2014, it twice provided aid packages worth more than $5.5 million to the three Ebola‐hit countries and Guinea‐Bissau. In comparison, large amounts of personnel and funding support from other countries did not arrive until after August and September, when EVD cases mushroomed and the potential global impact became clearer. Still, international pressures built with calls for China to play a more aggressive role in the global fight against Ebola. President Barack Obama voiced his frustration that China lagged behind the United States in funding the anti‐Ebola effort, while a UN agency criticized Chinese billionaires for not contributing enough to fight the virus.^47^

Did China contribute its fair share to the global fight against Ebola? It is hard to dispute that China trailed major OECD countries in certain aspects of health aid to Western Africa, such as building labs and ETUs. China constructed a total of 2 BSL labs, compared with 12 by the United States, 16 by Canada, and 3 by United Kingdom. It had only organized 1 ETU with 100 treatment beds, compared with 15 by the United States with 1700 treatment beds.^48^ Its share in global humanitarian funding against EVD outbreak was not significant, either. According to the data compiled by Office for the Coordination of Humanitarian Affairs (OCHA), in 2014 China contributed a total of $47 million (1.3% of the grand total), which was dwarfed by the $1.8 billion U.S. contribution (49% of grand total). To be sure, funding from China was higher than traditional donors such as Norway and Switzerland, as well as countries such as India and Russia. But China also lagged behind United Kingdom, Germany, the World Bank, the European Commission, France, Sweden, Japan, Canada, and the Netherlands in terms of total funding (**Table** **1**).

**Table 1:** Total humanitarian funding per donor to the EVD outbreak in 2014

Donor	Funding [million USD]	Grand total [%]
United States	1763	48.7
United Kingdom	364	10.1
Private (individuals and organizations)	200	5.5
Germany	167	4.6
World Bank	140	3.9
European Commission	119	3.3
France	108	3.0
Sweden	87	2.4
Japan	79	2.2
Canada	78	2.2
Netherlands	73	2.0
China	47	1.3
African Development Bank	46	1.3
Norway	41	1.1
Switzerland	38	1.1
Denmark	26	0.7
Belgium	22	0.6
Russia	21	0.6
India	11	0.3
Grand Total:	3618	100

Source: Financial Tracking Service, http://fts.unocha.org (Table ref: R24).

**Figure 1:**
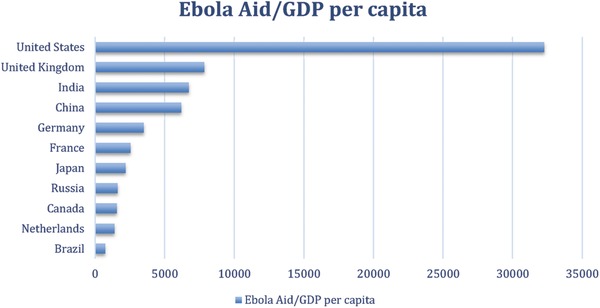
Aid amount/GDP per capita (in 2014 current US$) per capita ratio. Source: Aid data is taken from the financial tracking service (FTS) total funding per donor as of 20 February 2016 (http://fts.unocha.org (table ref: R24)); GDP data is taken from the World Bank, GDP/capita 2011–2015. http://data.worldbank.org/indicator/NY.GDP.PCAP.CD.

Total humanitarian funding as compiled by OCHA nevertheless may not be an accurate indicator of China's actual contribution to the global campaign against the EVD outbreak. China's contribution becomes much more impressive if adjusted according to gross domestic product (GDP) per capita. Adjusting for GDP per capita takes into account the country's still daunting domestic development challenges and its large population as compared to other OECD countries. When adjusted by GDP per capita, China's contribution is still no match of that of United States, but is close to that of United Kingdom, and higher than the contribution of other OECD countries. Interestingly, China and India had similar aid amount/GDP per capita ratio, suggesting a similarity in the dilemma that the two countries face in balancing their domestic development needs and the growing demands for them to assume more international responsibilities (**Figure** **1**).

Furthermore, the OCHA data does not include all in‐kind support such as ambulances, pickup trucks, motorcycles, incinerators, and personal protective equipment. It also does not include the contribution of medical staff and public health experts for Ebola containment efforts, which accounts for a significant share in China's humanitarian assistance. Equally important, it does not do justice to the shifting modalities of China's health aid to Africa. For a long time, the Chinese medical teams (CMTs) posted in Africa on a rotation basis have been the main instruments of China's health‐related development assistance in the region. While the CMTs play an instrumental role in providing routine medical services to local residents—indeed, a CMT treated the first case of Ebola in Conakry (Guinea's capital)—they are not adaptable and flexible enough to respond to large‐scale disease outbreaks. This may explain why in addition to mobilizing CMTs already on the ground, China also dispatched emergency medical teams whose members were recruited directly from domestic health institutions. In addition, while Beijing continued to rely on more traditional humanitarian assistance instruments such as in‐kind contributions and food aid, it also diversified the modalities of aid to building labs and treatment centers and health personnel training. This points to “a much more comprehensive, demand‐driven response” to affected countries' needs.^44^ In doing so Beijing might still prefer bilateral aid, but a significant volume of its humanitarian aid has been channeled through multilateral international institutions, suggesting that Beijing is becoming more flexible in backing coordinated international response mechanisms.^44^

## 5 Implications for Global Governance for PHEIC

A lot of ink has been spilled on the implications of the 2014 EVD outbreak for international relations and global governance.^49^, ^50^ Interestingly, very little has addressed China's role in the global fight against Ebola. This paper argues that China's participation sheds lights on its future engagement in global governance for public health emergencies. First, China's rise in a globalized context has made it increasingly sensitive to exogenous shocks such as PHEIC. While China's growing interaction with the outside world makes it more susceptible to global health hazards, its reemergence as a global power with ever greater interests also means that it cannot fulfill its domestic needs without a more proactive global strategy. In the case of Ebola outbreak, the risk of the virus infiltrating China, and evolving into a major outbreak was comparatively low. However, the sheer prospect of having imported cases wreak havoc on major international events China was to host, coupled with China's significant economic and political ties to Africa, prompted Beijing to support a swifter and more decisive approach to the EVD outbreak. It also explains why China quickly scaled up its efforts after it came under fire for the level of its response to the Ebola crisis. In responding to the criticism, China made it clear that its aid to Western Africa would not stop as long as the epidemic raged.^51^

Second, Beijing's prompt response to the Ebola crisis opens space for further international cooperation over global health security. Over the past two decades, the interconnection between global health and security has attracted attention from both policy makers and scholars alike.^52^, ^53^, ^54^, ^55^ Unlike the United States and other countries, Beijing has not explicitly framed the Ebola outbreak as an international security threat or deployed a large number of military personnel to the affected countries (as the U.S. did). Its dispatch of elite PLA units to the affected countries nevertheless suggests that it did view the outbreak as an existential security threat that required a response out of the normal political boundaries. Indeed, beginning in 2004 the PLA has been entrusted with “new historic missions” requiring it to increase its involvement in more straightforward humanitarian and relief operations.^56^ In a move that demonstrated its new power projection capabilities, it took the PLA No. 302 Hospital just 3 days to assemble a team of 31 medical personnel and mobilize 150 tons of material supplies for the mission to West Africa in September 2014. It took the PLA medical support forces in Sierra Leone just one week to convert a small general hospital into one specializing in treating infectious diseases, and just one month to construct a state‐of‐the‐art Ebola treatment centers (ETC) with 100 treatment beds. While there is no indication that PLA medical corps worked closely with their U.S. and U.K. counterparts in EVD diagnosis, treatment and containment, in light of the combined international efforts in this regard Chinese actions should be viewed as part of a larger effort by the richer countries to provide direct aid through personnel and materials.^57^ Furthermore, Beijing's willingness to implicitly securitize trans‐border disease outbreaks has opened a new area for future collaboration between China and other countries (e.g., the U.S.) under the Global Health Security Agenda, a global partnership that seeks to elevate global health security as a national and global priority. Indeed, during the crisis Chinese military personnel trained a Liberian engineering company so that the latter could play an instrumental role in helping the U. S. Army to construct its treatment center in the country. Similarly, the U.S. Air Force provided large forklifts to help unload the supplies that China brought to Liberia.^66^ When a limited supply of ZMapp (an experimental drug being tested against Ebola) was quickly exhausted, a small private Chinese company raced ahead in the fall of 2014 and within three months made more potentially lifesaving treatments available by producing about 100 doses of MIL77 based on information in ZMapp's patent.^58^ Meanwhile, the application of a security approach to the Ebola outbreak also underscored the need to reexamine the appropriateness of quarantine measures in handling acute disease outbreaks in the future. Like China, the Liberian government sought to enforce strict quarantine measures and restrict the movement of people during the crisis. While it is still unknown whether this actually resulted in reduced Ebola infections throughout the country, evidence did support the exacerbated conditions in the sealed districts.^59^ In that sense, Beijing's contribution to the fight against EVD in West Africa fits with Hedley Bull's model of a 'society of states' coming together to deal with a global public bad.^60^

Third, China's differentiated response toward the outbreak highlighted the challenges of reconciling the gap between domestic and foreign policy objectives. In view of the devastating power of the virus as well as the absence of core surveillance and response capacities in the three affected countries, the seemingly self‐serving containment strategy contributed positively to the global efforts to stem the spread of the virus and mitigate its devastating impact in West Africa. Its unprecedented generosity and its decision not to evacuate its citizens from affected countries may also help China win hearts and minds in the region. But the emphasis on preventing the virus from entering China also led China to discriminate against travelers from affected countries, such as imposing restrictions from athletes who were about to compete in the Youth Olympics. Unhappy about being stigmatized over fears of Ebola in China, Sierra Leone and Liberia decided against sending delegations to Nanjing. Nigeria did send its team, but it pulled out of the competition after its athletes were isolated and barred from training due to concerns over Ebola.^61^ While China might consider its discriminatory move necessary to protect its own people and ensure success of the games, it also tarnished its reputation and undermined its foreign policy objective of projecting soft power in the region.^62^ China faced a similar dilemma when dealing with the 2009 H1N1 pandemic, where government quarantine measures targeting Mexican citizens caused a diplomatic row between the two countries.^25^ This gap between domestic politics and foreign policy objectives is also attested to by the general absence of Chinese civil society organizations in international humanitarian efforts, even though a small number of Chinese professional volunteers reportedly participated in the front‐line fight against Ebola by joining MSF and other non‐governmental organizations (NGOs).^13^

Finally, while the crisis called for a coherent and coordinated approach to an overseas disease outbreak, the bifurcation of foreign policy and business interests in a new, globalized context makes such coordination increasingly difficult. In order to maintain laboriously built goodwill and future business opportunities in the region, it was in Beijing's foreign policy interest to distinguish itself from Western countries (whose workers fled West Africa when Ebola struck) by not evacuating the thousands of imported Chinese workers at sites.^63^ However, Ebola's devastating impact simultaneously triggered panic among Chinese nationals in the region. Not surprisingly, even though Beijing did not broadcast any evacuations, the number of Chinese living in affected countries—which included both workers affiliated with state owned enterprises and those who came there entirely of their own initiative—dropped by nearly half in the region from a high of 20 000 Chinese nationals in the summer of 2014.^64^ This forced the Chinese ambassador to Liberia to intervene and requested local Chinese companies not to allow their workers to leave the work sites.^23^ Moreover, as the state expanded its engagement in global anti‐Ebola efforts, China's private sector contribution remained negligible, especially in light of the fact that the number of billionaires in the country is second only to the United States. A UN agency reportedly lashed at out China's billionaires for not contributing enough to fight Ebola. In Liberia, locals were disappointed with the lack of significant contributions made by a Chinese mining company.^64^

## 6 Conclusion

During 2014–2015, China demonstrated a strong commitment to EVD prevention and control in West Africa. Through bilateral and multilateral channels, China kicked off its largest ever humanitarian mission in addressing a PHEIC. Its health aid package included cash and in‐kind contributions, the dispatch of a large number of health personnel, the construction of BSL labs and treatment centers, and the development and implementation of medical countermeasures. The unprecedented generosity served the domestic needs to prevent EVD from spreading into China, but it was also consistent with China's foreign policy objective to pursue soft power in Africa. While its total funding to EVD control in West Africa was no match of top donors like the United States, it becomes much more impressive when adjusted for GDP per capita. As Beijing becomes more sensitive to disease outbreaks overseas and as the scope of its humanitarian engagement grows and diversifies, the space for China's cooperation with international actors over global health security issues will only expand. The depth and breadth of that cooperation though will continue to be constrained by the gap between its domestic and foreign policy objectives, and the bifurcation of foreign policy and business interests overseas. In short, China's response to the EVD crisis in West Africa has revealed both opportunities and limits for its participation in future global governance for PHEIC.
